# Pathways to Children’s Behavioral Problems during the COVID-19 Pandemic: Fathers’ Parenting Stress and Parenting Approaches

**DOI:** 10.3390/children10040639

**Published:** 2023-03-29

**Authors:** Fatma Ozge Ünsal, Ibrahim Hakki Acar

**Affiliations:** 1 Department of Early Childhood Education, Faculty of Education, Goztepe Campus, Marmara University, 34722 Istanbul, Turkey; 2 Department of Psychology, Faculty of Social Sciences, Çekmeköy Campus, Ozyegin University, 34794 Istanbul, Turkey

**Keywords:** fathers, externalizing behaviors, internalizing behaviors, parenting stress, parenting approaches

## Abstract

Although the family stress model theoretically focuses on the roles of both mothers and fathers as predictors of children’s outcomes, studies generally have focused on mothers. The pandemic has brought additional burdens to parents’ daily functioning, including fathers’ involvement in childcare. The current study aimed to examine the contributions of fathers’ parenting stress and parenting approaches to their children’s behavior problems during the COVID-19 pandemic. Particularly, we examined the indirect effects of parenting stress on children’s behavior problems via parenting practices. The participants were 155 fathers (Mage = 36.87, SD = 5.11) and their children (71 girls, 84 boys; Mage = 59.52, SD = 14.98) from Turkish contexts. The fathers reported their parenting stress, approaches, and children’s behavioral problems. The results from the path analysis showed that parenting stress predicted children’s internalizing and externalizing behaviors. Parenting stress also predicted severe punishment and obedience as parts of the parenting approach. Finally, parenting stress was indirectly related to children’s externalizing behaviors via the punishment-based parenting approach of fathers. The findings of the current study highlighted the importance of examining the roles of fathers during the COVID-19 pandemic. Intervention programs targeting reducing fathers’ parenting stress and negative parenting approaches would also be beneficial for reducing children’s behavioral problems.

## 1. Introduction

Children begin their socialization within the immediate family environment. Family relationships are the main drivers of children’s social outcomes, including behavior problems. As part of the relationship dynamics, fathers have differentiated roles and effects on children’s outcomes [1]. Unprecedented events such as the COVID-19 pandemic could burden parents with young children, including fathers, leading them to have disruptions in their parenting [2,3]. External stressors due to the pandemic, such as financial burdens, job loss, and governmental regulations, could undermine fathers’ parenting stress and approaches, leading children to display heightened behavior problems. From this point of view, the current study aims to examine the contributions of fathers’ parenting stress and parenting approaches to their children’s behavior problems during the COVID-19 pandemic.

### 1.1. Family Stress Model: Fathers and Children during the COVID-19 Pandemic

The family stress model posits that economic and environmental stressors that emerge during unprecedented periods (e.g., the COVID-19 pandemic) can undermine parenting efficacy and approaches, leading children to be adversely affected by a lack of positive parenting or heightened negative parenting [3,4]. In addition, the family stress model emerged during the Great Depression in the United States and aimed to explain how parents, particularly those living in impoverished contexts, were influenced by the external stressors due to the Great Depression [5]. Recent work has transferred the family stress model to the COVID-19 pandemic process to explain how external stressors such as financial difficulties, job loss, and work–home balance disrupt family dynamics, including parenting [6,7].

Aligned with the classical conceptualizations of the family stress model, examining the adverse effects of the COVID-19 pandemic (e.g., financial struggles or job loss) could spill over to family dynamics such as escalated negative parent–child interactions and child behavior problems [8,9,10]. A recent conceptualization of the family stress model argues that perceived worry and stress by parents during the pandemic could yield similar cascading effects to financial burdens [6,10,11]. Browne et al. [6] present the COVID-19 Family Disruption Model, which is an adaptation of the family stress model to pandemic-related struggles. Considering the concepts of spillover or cascading effects of the family stress model, the COVID-19 Family Disruption Model posits that disruptions during the COVID-19 pandemic would be associated with increased stress in individuals, spouses, and the entire family, which would undermine reciprocal interactions between parents and children. For example, it was found that maltreatment of children increased due to escalated parent stress and disrupted family dynamics following the 2008 global recession [12]. In addition, Browne et al. [6] provide empirical evidence for the COVID-19 Family Disruption Model, showing that family dysfunction and children’s mental health were higher when the COVID-19 disruption (e.g., financial, family welfare, and household responsibilities) was higher, consequently predicting caregivers’ mental health and parenting quality over time. Overall, pandemic-induced stressors such as difficulties in homeschooling, escalated parenting tasks, or disrupted family dynamics contribute to the inefficacy of parenting [13]. Consequently, disrupted parenting practices during the COVID-19 pandemic could undermine children’s behavioral outcomes, including behavior problems [13]. Although the current study does not include all the family members in the investigation, the existing literature clearly suggests that reciprocal interactions among all the family members provide a context in which children develop competencies [14,15]. From this perspective, one should consider a triadic structure (father, mother, and child) when examining child outcomes within the family context.

### 1.2. Fathers’ Parenting Stress, Parenting Approaches, and Children’s Behaviors Problems

Children grow up in a socially complex ecological context where fathers and mothers influence each other’s parenting and their children’s well-being. Nevertheless, researchers mainly focus on mother–child interactions or relationship quality when predicting child outcomes [16,17,18]. Despite a decent amount of research, fathers have been overlooked as contributors to children’s development. However, revealing the differentiated roles of fathers in children’s development underscores the complexities of family dynamics, particularly during unforeseen periods such as the pandemic. Considering families as an integrated system of subsystems, including fathers, and understanding the roles of fathers in terms of their stress and parenting approaches in predicting children’s behavioral adjustment will substantially expand the previous research. Developmental models such as the Ecology of Father–Child Relationships posit that children are active agents of their development and that fathers’ parenting approaches directly or indirectly contribute to the children’s outcomes [19]. Although fathers’ parenting roles increased during the COVID-19 pandemic, gender disparity was reported regarding the parenting time allocated for educational activities and caregiving roles [20,21]. Nevertheless, fathers were more involved in childcare and household finances, especially when mothers lost their jobs during the pandemic [22]. External stressors such as unemployment decreased the co-parenting quality of both mothers and fathers and increased their parenting stress [23,24]. Previous empirical and conceptual work has provided mixed results, suggesting that fathers and mothers could be similarly affected by stressors or that fathers are more susceptible to stressors such that their parent–child relationships could be adversely affected [25,26,27,28]. Based on the aforementioned insights, we aimed to understand fathers’ inhibiting or accelerating roles by using parenting approaches to predict children’s behavior problems during the pandemic.

The pandemic process has had a negative impact on parents with young children. The primary external stressor that the pandemic brought into the family was the financial burden, which undermined the dynamics within the family [29]. Furthermore, financial instability or the risk of losing a job may enhance fathers’ parenting stress. Parenting stress is defined as a psychological burden that emerges due to the discrepancy between the needs and resources of parents regarding childcare [30]. Parenting stress could negatively affect the parent’s attitudes toward the child and the quality of their interactions [3,31]. The evidence is clear now that the pandemic escalated parenting stress due to difficulties in arranging the work–home balance and disrupted family dynamics [3,32]. The COVID-19 pandemic may have intensified unequal employment arrangements between fathers and mothers, leading to gender inequality at home such that mothers are more involved in childcare than fathers [33]. Nevertheless, parents, including fathers, particularly those living in worsened conditions such as job loss, experienced depression and consequently displayed harsh parenting and negative parent–child interactions [32,33,34,35]. Heightened parenting stress could be an adverse undermining factor for positive parenting practices and children’s behavioral adjustment [36].

Changes that emerged during the pandemic also adversely affected parenting efficacy and approaches [21,32]. These results suggest that parenting stress could undermine positive parenting practices and escalate negative parenting practices. Parenting approaches can be categorized into two groups. First, positive parenting approaches consist of inductive reasoning and warmth, reflecting mutual attunement and nurturing involvement. Negative parenting practices include obedience and punishment, reflecting the struggle between parents and children [37]. Obedience, as a negative aspect of parenting, includes parents’ demands and expectations regardless of children’s needs (e.g., a parent requires a child to do what s/he is told without questioning). Punishment contains negative psychological and physical approaches toward children (e.g., forbidding a child from doing what s/he likes when s/he misbehaves or slapping a child to redirect her/his behavior). In several studies, positive parenting practices have been associated with fewer behavior problems, better adjustment, positive peer relations, and prosocial behaviors [38,39,40,41].

On the contrary, negative parenting practices have been found to be related to internalizing and externalizing behavior problems in children [38,42,43,44]. Due to the changes that emerged during the pandemic, fathers’ parenting practices may have been adversely affected. For example, fathers who are financial providers for the household could feel emotionally and physically drained, meaning they may experience escalations in their negative parenting practices and a reduction in positive parenting practices [3]. Explaining how the parenting approaches of fathers predict children’s behavior problems during the pandemic will provide insights into how fathers’ parenting practices uniquely contribute to children’s behavior problems.

### 1.3. Bringing It All Together: Indirect Effects of Parenting Stress on Children’s Behavior Problems via Parenting Practices

The family stress model posits that children’s behavior (such as behavior problems) is affected by changes in how parents handle the stress that comes from outside sources. The COVID-19 pandemic has brought unprecedented adversity that has escalated the parenting stress of parents, including fathers, undermining positive parenting practices [3]. Consequently, disrupted parenting approaches, reflecting negativity and/or a lack of positive parenting, heighten children’s behavior problems. Fathers may feel a lack of resources due to experiencing higher levels of parenting stress that undermines their parenting practices and causes them to approach their children from negative perspectives, triggering their children’s behavior problems. Overall, in the context of fathers with heightened parenting stress, leading to negative parenting practices, children may display higher levels of behavior problems. From this point of view, understanding the link between parenting stress and children’s behavior problems through parenting approaches will uncover complexities in the context of fathers as part of a family.

### 1.4. Current Study

Although the family stress model theoretically focuses on the roles of both mothers and fathers as predictors of children’s outcomes, studies generally have focused on mothers. The pandemic has brought additional burdens to parents’ daily functioning, including fathers’ involvement in childcare. These burdens may have undermined the capability of fathers’ parenting approaches to their children due to escalated parenting stress and juggling the home–job balance. To the best of our knowledge, no study has examined the roles of fathers’ parenting stress and approaches to children’s behavior problems during the COVID-19 pandemic. A large body of research investigated the roles of mothers in childcare during the COVID-19 pandemic [2,3,45]. In the current study, Turkish fathers, as understudied agents within the family context, are considered the primary driver of children’s behavioral problems in family contexts. The current study aimed to examine the contributions of fathers’ parenting stress and parenting approaches to their children’s behavior problems during the COVID-19 pandemic. Particularly, we examined the indirect effects of parenting stress on children’s behavior problems via parenting practices. We hypothesized that higher levels of a father’s parenting stress would be positively related to higher levels of a child’s behavior problems. In addition, heightened parenting stress would positively predict negative parenting approaches (i.e., obedience and punishment) and negatively predict positive parenting approaches (i.e., inductive reasoning and warmth), predicting higher levels of behavior problems in children.

## 2. Materials and Methods

### 2.1. Participants

In this cross-sectional study, 155 Turkish fathers and their children (71 girls and 84 boys) were recruited. The children’s ages ranged from 15 to 98 months, with a mean of 59.52 months and a standard deviation of 14.98 months. During the data collection, 97.4% of the fathers and 45.2% of the mothers were employed. The fathers’ ages ranged from 27 to 56 years, with a mean of 36.87 years and a standard deviation of 5.11 years. The mothers’ ages ranged from 24 to 49 years, with a mean of 33.29 years and a standard deviation of 4.40 years.

The chain referral sampling technique was utilized to recruit participants, starting by contacting fathers in the immediate circle of the researchers and then expanding the recruitment pool through referrals from the recruited fathers. A composite variable for socioeconomic status was created by averaging the standardized (z-score) levels of the fathers’ education and income.

### 2.2. Procedure

The Institutional Review Board (IRB) at Marmara University approved the study with approval number 254368 on 21 March 2022. The study was announced on social media, and everyone in the researchers’ immediate circle was given the link to the questionnaires and consent form. The announcement told people who might want to take part in the study what it was about and what it was trying to do. It also stressed that taking part was voluntary and anonymous. The online survey was in Turkish. We did not provide incentives for participation. Before administering the survey, the participants confirmed their voluntary participation by clicking a predesigned checkbox on the form, indicating informed consent. Data were collected between March and May 2022. During the data collection, although there were some changes in the restrictions, some of the rules for the pandemic, such as social distancing, wearing masks indoors, and distance education, were continued.

The participants completed the questionnaires using Google Forms, which took approximately 15 minutes. The study’s inclusion criteria were that the participants should be fathers with at least one young child. All the collected data were stored on a password-protected computer accessible only to the authors, ensuring participant confidentiality.

### 2.3. Measures

The survey completed online consisted of four sections: demographic information, the Parenting Stress Questionnaire, the Child Rearing Questionnaire, and the Strengths and Difficulties Questionnaire.

#### 2.3.1. Fathers’ Parenting Stress

The Parenting Stress Scale (PSS) [46] was used to assess the fathers’ stress levels. The PSS, which measures psychological stress related to the responsibilities of the parenting role, consists of 18 items (e.g., “*I regret being a parent*”). The fathers reported their parenting stress levels on a 5-point Likert-type scale ranging from 0 (definitely does not describe me) to 4 (definitely describes me). The PSS has been developed, used, and validated in Turkey [46]. The internal reliability of the PSS in this study, as measured by Cronbach’s alpha, was 0.91.

#### 2.3.2. Fathers’ Parenting Approaches

The fathers reported on the Child-Rearing Questionnaire (CRQ) [37]. The CRQ is a 30-item scale that is rated on a 5-point Likert-type scale (1 = Never and 5 = Always). The CRQ has been validated and used with Turkish parents [44,45,46]. It has been validated and used with Turkish parents [47,48,49]. The CRQ has four subscales measuring warmth (e.g., “*My child and I have warm, intimate times together*”), inductive reasoning (e.g., “*I try to explain to my child why certain things are necessary*”), punishment (e.g., “*I use physical punishment, e.g., smacking, for very bad behavior*”), and obedience-demanding (e.g., “*I expect my child to do what he/she is told to do without stopping to argue about it*”). The internal consistency scores (Cronbach’s alpha) for inductive reasoning, punishment, obedience-demanding, and warmth were 0.76, 0.67, 0.78, and 0.81, respectively.

#### 2.3.3. Children’s Behavior Problems

To assess the children’s externalizing and internalizing problems, the Turkish version of the Strengths and Difficulties Questionnaire (SDQ) [50,51] was used. The SDQ has been shown to have high reliability and validity across cultures [42,51,52]. In line with previous research [41,51], emotional symptoms (e.g., *many worries, often seems worried*) were used to assess the children’s internalizing problems, while conduct problems and the hyperactivity subscales were used to evaluate the children’s externalizing problems (e.g., *often lies or cheats*). Each subscale includes 5 items on a 3-point scale ranging from 0 (not true) to 2 (certainly true). The sum of the items was used to create composite scores. Given that the distributions of the subscales were skewed and the scaling was ordinal, McDonald’s Omega was used to measure the reliability of the subscales rather than Cronbach’s alpha [53]. The reliability of the internalizing behavior subscale was 0.61, while the reliability of the externalizing behavior subscale was 0.71. We tested the common variance among the items for internalizing and externalizing behaviors using Harman’s single-factor test [54]. The results showed that only 20.24% of the variance was captured in the first unrotated factor (50%), indicating that common method variance was not an issue in the study [54].

### 2.4. Data Analysis

There were no missing data in the current study. However, as the data were collected from the same respondents using self-reported surveys at one point in time, common method bias may have been present [55]. To test for common method variance, Harman’s single-factor test was used as a post hoc analysis to see if a single factor could account for the variance in the data [54]. The results showed that only 14.81% of the variance was captured by the first unrotated factor (<50%), indicating that common method variance was not an issue in the study [54]. The skewness values of the variables ranged from 0.56 to 2.12, and the kurtosis values ranged from 0.081 to 5.39, indicating a multivariate normal distribution (skewness < 3 and kurtosis < 10) [56,57].

We used the bootstrapping method with 2000 resamples and 95% confidence intervals [58] to test for indirect effects. The bias-corrected bootstrap confidence intervals were used because they have been shown to test the significance of indirect effects more accurately than other methods [59]. A top-down model-building approach was used, where all the possible covariates were added to the models and then sequentially removed if they were not significant. Initially, the following covariates were included in the model: child’s age, sex, and father’s SES. The nested models were compared using the Δχ2 test [60], and the final parsimonious model was reported. Maximum likelihood estimation was used in *Mplus 8.4* [61]. According to Kline’s [57] recommendations, a good fit is defined as root mean square error of approximation (RMSEA) values of 0.08 or below, standardized root mean square residual (SRMR) values of 0.08 or below, and comparative fit index (CFI) and Tucker–Lewis index (TLI) values larger than 0.90.

## 3. Results

### 3.1. Preliminary Results

The bivariate correlations (Pearson) among the variables were calculated. The results showed that internalizing behaviors were correlated with inductive reasoning (*r* (155) = −0.22), warmth (*r* (155) = −0.16), and parenting stress (*r* (155) = 0.31). Externalizing behaviors were associated with inductive reasoning (*r* (155) = −0.22), obedience (*r* (155) = 0.27), punishment (*r* (155) = 0.31), and parenting stress (*r* (155) = 0.27). See Table 1 for the complete correlation results.

### 3.2. Structural Model

We examined the parenting approaches’ direct and indirect effects on the association between parenting stress and children’s behavior problems (externalizing and internalizing). The final structural model using manifest variables fit the data well: *χ^2^* (10) = 6.955, *p* = 0.72, CFI = 1.00, TLI = 1.00, RMSEA = 0.00 [90% CI: 0.000–0.06], SRMR = 0.03, AIC (Akaike Information Criterion) = 3071.087, BIC (Bayesian Information Criterion) = 3189.781.

There was a direct effect of parenting stress on internalizing (*β* = 0.25, 95% CI: 0.04–0.31) and externalizing behaviors (*β* = 0.20, 95% CI: 0.05–0.37). In addition, there was a direct effect of punishment on externalizing behaviors (*β* = 0.21, 95% CI: 0.02–0.35). Parenting stress was related to punishment (*β* = 0.24, 95% CI: 0.07–0.40) and obedience (*β* = 0.24, 95% CI: 0.10–0.38) as parts of the parenting approach.

**Figure 1:**
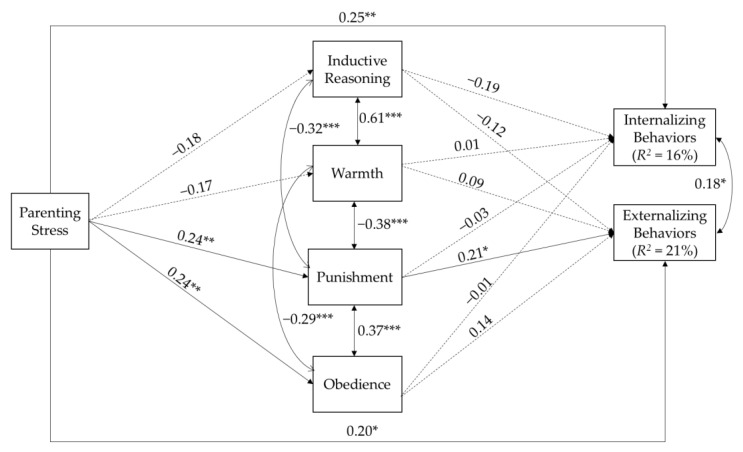
The path model with standardized coefficients. Fathers’ parenting stress predicting children’s internalizing and externalizing behaviors through parenting approaches. Note. Dashed lines represent nonsignificant paths. Child’s age as a covariate was controlled for the internalizing (*β* = 0.18, *p* = 0.01) and externalizing behaviors (*β* = −0.19, *p* = 0.01). *** *p* < 0.001, ** *p* < 0.01, * *p* < 0.05.

Parenting stress was indirectly related to children’s externalizing behaviors via the punishment-based parenting approach of fathers (*β* = 0.05, 95% CI: 0.01–0.11). Indeed, the results showed that fathers with higher levels of parenting stress showed higher levels of punishment in their parenting, which, in turn, predicted higher levels of externalizing behaviors. There was no other significant indirect effect. See Figure 1 for a depiction of the model.

## 4. Discussion

In the current study, we investigated the contributions of fathers’ parenting stress and parenting approaches to their children’s internalizing and externalizing behaviors during the COVID-19 pandemic. Fathers’ contributions to children’s outcomes have long been neglected in the literature, particularly in the Turkish parenting context (see [17,23,62,63] for international investigations). We, therefore, aimed to extend the previous work by examining the contributions of fathers’ stress and parenting approaches to children’s behavioral problems, particularly during the COVID-19 pandemic. The results of the current study showed that parenting stress and negative parenting approaches (e.g., obedience) were related to children’s behavior problems. Each finding is discussed in turn below.

First, we found that parenting stress was related to higher levels of punishment and obedience in their parenting. In detail, the fathers with higher levels of parenting stress utilized heightened punishment and obedient parenting approaches when taking care of their children. These findings are congruent with previous work [17,18,23,64,65,66], including parenting during the pandemic, showing that parents with excessive stress due to parenting tasks tend to employ negative parenting practices such as aggressive discipline and harsh parenting. Interestingly, contrary to previous work [23,24], parenting stress was not related to positive parenting approaches (i.e., inductive reasoning and warmth). The discrepancies between the current findings and previous research could be partly due to methodological differences; therefore, further research is warranted to explore these discrepancies. To the best of our knowledge, no study has examined the association between fathers’ parenting and parenting approaches in the Turkish parenting context in Turkey. Therefore, further studies are needed to understand this relationship.

Second, we found that parenting stress was related to children’s internalizing and externalizing behaviors. Children in the context of heightened parenting stress displayed higher levels of internalizing and externalizing behaviors. Aligned with previous studies [3,44], due to the pandemic there were increased demands on fathers to adapt to new home–work routines, including caring for their children. This increased burden and parenting stress could lead fathers to disrupt their relationships with their children; consequently, children may express behavioral problems. As explained in the next section, parenting stress could be reflected in parenting approaches, undermining children’s behavior problems.

Third, there was an indirect effect of parenting stress on children’s externalizing behaviors through a punishment-based parenting approach. The fathers with heightened parenting stress in the context of the COVID-19 pandemic employed punishment in their parenting, consequently undermining their children’s externalizing behaviors. The reason behind this finding could be explained from the perspective of the family stress model [22]. In detail, the fathers could not regulate their parenting stress and used aggressive discipline or harsh parenting toward their children. Consequently, the children exposed to harsh parenting displayed externalizing behaviors. Consistent with previous work [3,21,67], the results of the current study suggest that heightened parenting stress leads fathers to be more likely to engage in punishment in their parenting, which increases their children’s externalizing behaviors.

It is noteworthy that there was no indirect effect of parenting stress on internalizing behaviors through parenting approaches. There could be two explanations for this finding. First, the fathers did not report higher levels of internalizing behaviors for their children (M = 1.29; range = 0–6). The mediation mechanism through parenting strategies did not have an impact on those children who were believed to have low levels of internalizing behaviors. Second, aligned with the preceding explanation, the fathers were not able to capture their children’s internalizing behaviors [68].

It is worth discussing that fathers and mothers may have experienced different levels of parenting stress during the COVID-19 pandemic. Considering the culturally informed gender roles in the Turkish family context, mothers may carry more weight when it comes to child caring, and this may have increased mothers’ parenting stress more compared to fathers [69]. Furthermore, Cheung et al. [70] suggest that fathers’ stress levels are only related to their regulation, while mothers’ stress levels are related to their own and their child’s dysregulation. From this point of view, albeit it was not the focal purpose of the current study, one could say that with increased daily household chores, child caring, and dealing with children’s educational needs, mothers may have heightened parenting stress during the pandemic.

## 5. Limitations and Future Research

Even though this cross-sectional study adds to what is known about fathers and children during the COVID-19 pandemic, it is important to note that this study has some problems. First, the cross-sectional nature of the study limits making causal inferences. Future longitudinal data collection could assist in running pure mediation models. Second, the small sample size has prevented us from generalizing the findings to a larger population of fathers in Turkey. Possible measurement issues could be involved as the collection was based solely on self-reports. Dyadic data collection and observational methods could diversify the data collection and reduce the measurement error. Third, we should acknowledge that we did not assess the direct external economic hardship/pressure as part of the family stress model that emerged during the pandemic related to parenting and child outcomes in the current study. However, we used the family stress model to lay the foundation for reflecting changes in the fathers’ parenting approaches due to their parenting stress and their association with their children’s behavior problems. Fourth, considering that parenting approaches may be differentiated depending on the child’s age [71], the wide age range of children in the current study should be considered when interpreting the results. Finally, we acknowledge that parent–child interactions are not only dyadic but also triadic in nature. That includes the father, mother, and child within a family context. Therefore, future work should include the father, mother, and child in the investigation to provide a comprehensive and triadic-based structure of interactions.

## 6. Conclusions

In conclusion, fathers’ parenting stress is an important predictor of negative parenting styles (e.g., punishment and obedience) and children’s behavior. Parenting stress was indirectly associated with children’s externalizing behaviors through punishment-based parenting. Overall, the findings of the current study highlighted the importance of examining the roles of fathers during the COVID-19 pandemic. Intervention programs targeting reducing fathers’ parenting stress and negative parenting approaches would also be beneficial for reducing children’s behavioral problems.

## Figures and Tables

**Table 1:** Bivariate Correlations and Descriptive Statistics for the Study Variables.

Variables	1	2	3	4	5	6	7	8	9	10	11	12
1. Internalizing Behaviors	-											
2. Externalizing Behaviors	0.23 **	-										
3. Inductive Reasoning	−0.22 **	−0.22 **	-									
4. Warmth	−0.16 *	−0.14	0.62 **	-								
5. Punishment	0.10	0.31 **	−0.35 **	−0.41 **	-							
6. Obedience	0.12	0.27 **	−0.30 **	−0.32 **	0.41 **	-						
7. Parenting Stress	0.31 **	0.27 **	−0.18 *	−0.17 *	0.24 **	0.24 **	-					
8. Child’s Age	0.22 **	−0.11	−0.01	−0.07	0.09	0.14	0.17 *	-				
9. Mother’s Age	0.11	−0.03	0.01	−0.07	−0.12	−0.09	0.06	0.06	-			
10. Father’s Age	0.05	−0.07	0.05	−0.09	−0.10	−0.05	0.07	0.17 *	0.69 **	-		
11. Family’s SES	−0.17 *	−0.06	0.08	0.05	−0.03	−0.17 *	−0.35 **	−0.26 **	0.021 **	−0.02	-	
12. Child’s Sex	0.03	0.01	0.02	−0.06	0.02	−0.07	−0.08	−0.15	0.04	0.02	0.08	-
*n*	155	155	155	155	155	155	155	155	155	155	155	155
Mean	1.29	4.81	4.42	4.60	1.52	2.43	6.79	59.52	33.29	36.87	0.00	
SD	1.48	2.93	0.44	0.37	0.33	0.76	9.16	14.98	4.40	5.11	0.86	
Range	0–6	0–12	2.86–5	3.33–5	1–3.13	1–4.33	0–71	15–98	24–49	27–56	−2.14/2.64	

Note. * *p* < 0.05, two-tailed. ** *p* < 0.01, two-tailed. Sex: 1 = Girl, 0 = Boy. SES = Socioeconomic Status.

## Data Availability

The datasets generated during and/or analyzed during the current study are available from the corresponding author upon reasonable request.
